# Readmission Within the First Day of Discharge Is Painful: Experience From an Australian General Surgical Service

**DOI:** 10.7759/cureus.32209

**Published:** 2022-12-05

**Authors:** Rama Chidambaram, Sharie Apikotoa, Rhiannon Hicks, Mary Theophilus, Ruwan Wijesuriya

**Affiliations:** 1 General Surgery, St John of God Midland Public and Private Hospitals, Perth, AUS; 2 Vascular Surgery, Royal Perth Hospital, Perth, AUS

**Keywords:** healthcare costs, general surgery, failed discharge, readmission, pain

## Abstract

Background

Unplanned readmission to the hospital after discharge is a costly issue for healthcare systems and patients. It is a delicate balance between the resolution of the surgical problem and the length of hospital stay. Most studies have focused on readmissions within 28 or 30 days after discharge, despite data showing that many occur early in this period. This study examined the reasons for unplanned readmission within the first day after discharge.

Methods

A retrospective cohort analysis of readmissions between 1st May 2016 and 1st May 2021 was undertaken by chart review. Readmissions on the “day of” and the “day after” discharge and their respective index admissions were identified via the hospital’s patient administration database, webPAS (DXC Technology, USA).

Results

There were 126 readmissions (0.5%) across 25,119 admissions. Common reasons for readmission were pain (28%, n=35), readmission for the same diagnosis (21%, n=26), surgical site infection (SSI) (11%, n=14), bleeding (11%, n=14) and ileus (6%, n=7). Analysis of index admissions showed that 18/35 readmissions for pain had inadequate pain management based on pain scores, analgesic use and discharge medications and 7/14 readmissions for SSI did not have appropriate treatment of a recognised SSI or did not have antibiotic prophylaxis guidelines adhered to. Fourteen of 26 readmissions for the same diagnosis received just continuation of treatment initiated at index admission.

Conclusion

Pain is the most common reason for readmission within the first day after discharge in surgical patients. Better pain management, following antibiotic prophylaxis guidelines, and involving patients in discharge planning could prevent many readmissions.

## Introduction

Unplanned readmissions to the hospital soon after discharge are costly to healthcare systems and patients [1-3]. The additional cost of lost potential in treating other patients is also often not accounted for [2]. Recent Australian data show that unplanned readmissions are common and between 7.4% and 10.9% [4,5]. For patients, these readmissions are distressing, disruptive and lead to distrust in the healthcare system [3].

Despite the worldwide emphasis on reducing unplanned readmissions, most studies have examined readmissions in medical patients as opposed to surgical, despite differences between the two cohorts [2]. First, surgical patients are readmitted not only due to complications that arise from their medical comorbidities but also due to complications that are specific to their surgery [6]. Second, a proportion of surgical index admissions are for elective surgery, so preoperative optimisation of the patient poses a unique opportunity to prevent unplanned readmissions [2]. Third, while it remains controversial if readmission rates among medical patients equate to quality of health care, it has been shown that hospitals with high surgical volume and low surgical mortality have lower surgical readmission rates than others [7,8]. Further, few studies that do examine surgical unplanned readmissions are limited to an operation, e.g. total hip arthroplasty, or a process, e.g. day-procedures, or a patient population, e.g. patients with the peripheral arterial disease [9-11]. 

The overwhelming majority of studies in this field are also limited to examining unplanned readmissions within a timeframe of 28-30 days despite Australian data showing that approximately 10% of 28-day unplanned readmissions occurred within the first day of discharge [4,5,12]. Inter-hospital variation in readmissions is also greatest in the days just after discharge and early readmission has been suggested to be a better gauge for the quality of care provided by a hospital [13].

The aim of this study was to examine the reasons for unplanned readmission to an Australian General Surgical service within the first day of discharge. This is a critical step in developing strategies to reduce surgical unplanned readmission rates.

## Materials and methods

Design

The study was designed as a retrospective cohort analysis.

Setting

The setting was the General Surgical department at St John of God Midland Public and Private Hospitals, which is an outer metropolitan teaching hospital in Perth, Western Australia. The department provides all general surgical services including specialist colorectal, breast, endocrine and bariatric services. The department primarily services an adult population; however, patients between the ages of 13 and 18 are treated on a select basis. The hospital has an intensive care facility; however, patients with large blood products or tertiary-level care requirements are routinely transferred to the nearby tertiary referral centre. 

Definitions

A surgical admission was defined as any that resulted in a patient being admitted to the General Surgical inpatient service. The initial admission was termed the “index admission”.

Inclusions

Readmissions that occurred on the day of discharge or on the day after discharge were included. No exclusions were made on the basis of age, ambulatory versus multi-day index admissions, elective versus emergent index admissions and funding status of index admission and readmission.

Exclusions

Readmissions where patients had “discharged against medical advice” from their index admission, where the index admission was at another hospital, or where the patients were “stepped down” to another hospital or facility following their index admission were all excluded. Planned readmissions and readmission after “day leave" were also excluded.

Data collection

Data on admissions and readmissions on days 0 and 1 to the General Surgical service between 1st May 2016 and 1st May 2021 were extracted from webPAS (DXC Technology, USA), the hospital’s patient administration database. Patient age, length of stay for both index admission and readmission and source of index admission were recorded. A review of the medical record was conducted for all identified readmissions by two surgical registrars (RC and SA). For each identified readmission, the reasons for index admission and readmission and an analysis of whether adequate steps to prevent readmission had been taken at index admission were recorded.

Statistical analysis

Descriptive statistics were used to analyse data. Specifically, categorical data were expressed as a percentage, continuous data that were normally distributed were expressed as mean ± standard deviation (SD) and continuous data that were non-normally distributed were expressed as median ± interquartile range (IQR). Tabulation of results was created on Word (Microsoft, USA).

Ethics

The study was conducted as part of the ongoing departmental audit and quality improvement. All data were completely anonymised and there were no direct or indirect identifiers of human data. The principles outlined in the Australian Privacy Principles, the Declaration of Helsinki regarding human experimentation and the 2015 SQUIRE Guidelines in reporting on quality improvement were strictly adhered to. 

## Results

Between 1st May 2016 and 1st May 2021, there were 25,119 admissions to the General Surgical service. Of these, 141 admissions were associated with a readmission that occurred on the day of discharge or on the day after discharge. After applying the exclusion criteria, 15 were voided, resulting in 126 readmissions (0.5%). The mean (±SD) age of patients being readmitted was 52 (±20) years. The median (IQR) length of stay of index admissions was 2 (1-4) days, and of readmissions was 2 (1-4) days.

Of the readmissions, 39 (31%) were following admission for elective surgery and 87 (69%) were following admission for emergent General Surgical presentation. Of those who were originally admitted emergently, 38 out of the 87 (44%) received surgery during their index admission while the remaining 49 out of 87 (56%) received conservative management during their index admission. 

The eight most common reasons for unplanned readmission in the cohort were as shown in Table 1. The most common reason was pain (28%) and featured prominently as a reason for readmission regardless if the index admission was elective or emergent or involved surgery. Other common reasons included surgical site infection (SSI) (11%), bleeding (11%) and ileus (6%). The least common reasons were grouped into the category “Other” and included post-operative nausea and vomiting, constipation, choledocholithiasis and social issues.

**Table 1:** Common reasons for readmission to hospital within one day of discharge from a General Surgical service SSI, surgical site infection; HAP, hospital-acquired pneumonia; VTE, venous thromboembolism.

	Pain	Same reason as index admission	SSI	Bleeding	Ileus	Leak	HAP	VTE	Other	Total
Overall	35	26	14	14	7	4	2	2	22	126
Readmission following conservative management in emergent index admission	17	26	0	0	0	0	1	1	4	49
Readmission following surgery during emergent index admission	10	0	10	3	4	1	0	0	10	38
Readmission following admission for elective surgery	8	0	4	11	3	3	1	1	8	39

The second most common reason for readmission was readmission for the same diagnosis as was made in the index admission. This was the cause for readmission in 26 patients and they had all been conservatively managed during their index admission. Most of this group received either “continuation of the treatment they had originally received” (n=14) or “escalation of treatment” (n=8). However, a change in diagnosis was made in four patients and, consequently, a change in treatment. Switching from oral to intravenous antibiotic therapy and any procedural intervention, e.g. percutaneous abscess drainage, was considered an escalation of treatment. 

The reasons for readmission in those who received surgery during their index admission were categorised according to the Clavien-Dindo classification to measure the severity of the reason for readmission (Table 2). No patient was readmitted for a life-threatening complication.

**Table 2:** Clavien-Dindo classification of reasons for readmission following surgery during index admission

	Grade 1	Grade 2	Grade 3a	Grade 3b	Grade 4a	Grade 4b	Grade 5	Total
Readmission following conservative management in emergent index admission	18	12	3	5	0	0	0	38
Readmission following surgery during emergent index admission	24	9	0	6	0	0	0	39

The proportion of readmission that required a procedural intervention was 22% (n=28). This included surgery, percutaneous abscess drainage and bedside wound debridement but did not include placement of an intravenous catheter or urethral indwelling catheter. The distribution of those requiring a procedural intervention across elective, emergent conservatively managed and emergent surgically managed groups and across the reason for readmission is shown in Table 3.

**Table 3:** Reasons for readmissions requiring procedural intervention distributed across nature of index admission SSI, surgical site infection; HAP, hospital-acquired pneumonia; VTE, venous thromboembolism.

	Pain	Same reason as index admission	SSI	Bleeding	Ileus	Leak	HAP	VTE	Other	Total
Readmission requiring procedural intervention following conservative management in emergent index admission	5	10	0	0	0	0	0	0	0	15
Readmission requiring procedural intervention following surgery during emergent index admission	0	0	4	1	0	0	0	0	1	7
Readmission requiring procedural intervention following admission for elective surgery	0	0	2	1	0	3	0	0	0	6

In an examination of whether adequate steps were taken to prevent readmission, it was found that across the whole cohort, 51% of those readmitted for pain (n=35) were deemed not to have had adequate pain management, while the remaining 49% were deemed to have had this. In determining this, day-of-discharge pain scores and analgesic requirements and adequacy of discharge analgesia were assessed. The distribution of those managed adequately versus inadequately was similar across elective, emergent conservatively managed and emergent surgically managed groups (Table 4).

**Table 4:** Distribution of adequacy in pain management in readmitted patients across nature of index admission

	Inadequate pain management	Adequate pain management
Overall	18	17
Readmission following conservative management in emergent index admission	7	10
Readmission following surgery during emergent index admission	6	4
Readmission following admission for elective surgery	5	3

SSI was considered inadequately managed if local surgical antibiotic prophylaxis guidelines had not been adhered to or if the duration or delivery of antibiotic therapy for an SSI identified in the index admission was considered inappropriate. Fifty percent of SSIs (n=14) across the whole cohort were found to be managed inadequately and the distribution across those who received surgery during their index admission is shown in Table 5. 

**Table 5:** Adequacy of peri-operative SSI management in those who were readmitted following surgery during their index admission SSI, surgical site infection.

	Inadequately managed SSI	Adequately managed SSI
Overall	7	7
Readmission following surgery during emergent index admission	4	6
Readmission following admission for elective surgery	3	1

Anticoagulation status was reviewed for those readmitted for bleeding (n=14) and the large majority were only on prophylactic levels of anticoagulation (Table 6). Only one patient who had elective surgery and one patient who had emergency surgery in their respective index admissions required surgery during their readmission to manage bleeding. Blood products were administered during readmission to one patient who had elective surgery in their index admission. The remainder were all conservatively managed for their bleeding during readmission. 

**Table 6:** Anticoagulation status for patients who were readmitted for bleeding following surgery during index admission

	Therapeutic anticoagulation	Prophylactic anticoagulation	No anticoagulation
Overall	2	11	1
Readmission following surgery during emergent index admission	0	3	0
Readmission following admission for elective surgery	2	8	1

## Discussion

Data on readmission rates within the first day after discharge are scarce but may be estimated to be around 1% of all admissions [4,5]. This was in line with our results of 0.5%. The scale of this issue is only apparent when it is appreciated that the number of patients discharged in Australia in 2017-2018 was 11.2 million, so approximately 100,000 patients each year may be being readmitted within the first day after discharge [14].

Readmissions in our cohort were skewed towards patients who had originally been admitted for an emergent presentation and this has been corroborated in numerous medical and surgical cohorts [2,4,15]. Emergent admissions are usually longer than elective admissions and this could increase the exposure to nosocomial bacteria and, consequently, the risk of perioperative infections [2]. This is supported by the findings that show that the length of stay of index admission predicts the risk of readmission [15].

The pain was the most common reason for readmission and affected 28% of our cohort. Unfortunately, pain following discharge is prevalent in both medical and surgical patients [16,17]. It has also been well described that this pain leads to readmissions [10,15,18]. A systematic review examining General, Colorectal, Bariatric and Vascular surgical readmissions found that eight out of 31 studies ranked pain as one of their top three reasons for readmission [15]. The same review also found pain to be a more common reason for readmission amongst General and Bariatric surgical readmissions [15]. Australian data from Victorian medical and surgical readmissions within the first day after discharge found 26.3% of 162 unplanned readmissions were due to poor pain management, and it closely reflects our results [12]. The proportion of very early readmission due to pain is likely underrepresented and masked in the current literature which largely consists of analyses of readmissions 28-30 days after discharge.

Patients suffering pain after discharge and after surgery have been shown to suffer low quality of life related to impaired social, mental and physical function [16]. A study examining pain within 24 hours of the day of surgery found that unrelieved acute pain impacted sleep and this consequently affected physical function [19]. The effects are prolonged, and functional impairment caused by severe acute postoperative pain has been shown to last six months after surgery [20]. There is also increasing evidence of the association between the level of acute pain and the risk of developing chronic pain and this has been observed in surgical and medical patients [21]. There may be thus two-fold economic benefit in treating acute postoperative pain: first, in reducing costs from reduced recovery time, length of stay and readmissions; and second, in reducing the economic burden related to the treatment of chronic pain [10,22].

Patient interviews report a heightened awareness and anxiety surrounding pain post hospitalisation and provides an important perspective as to why readmission for pain is so frequent [23]. Patient and clinician concerns surrounding the prescription of opioid medication may also play a role in pain management on discharge [24]. Importantly, pain scores in combination with analgesic use on discharge have predicted unplanned readmission at seven and 30 days after discharge [18]. This is in line with our findings which reflect that close to 50% of pain-related readmissions within the first day after discharge may have been prevented by better pain management on the day of discharge. Targeting better pain management on the day of discharge should be the first step towards reducing pain-related readmissions and towards improving multiple patient outcomes. 

The second most common reason for readmission in our cohort (21%) was for the same diagnosis as was made during the index admission. It was a prevalent reason for readmission in the literature and other studies reported that between 44% and 71% of readmissions were for the same reason as the index admission [24]. In our cohort, it was only associated with patients who were conservatively managed for emergent presentations and more than half of this group were readmitted for this reason. Australian data on medical and surgical patients also reported that a high proportion of readmissions within the first day after discharge were associated with the reason for index admission [12]. Inadequate treatment of the index admission illness and premature discharge have been recognised as factors leading to these readmissions [25]. This is supported by our results which found that more than half of the patients who were readmitted for the same reason as their index admission, received just a continuation of their original treatment. Unfortunately, due to the limited resources of all healthcare systems, clinicians face significant pressures to reduce the length of stays and to shift non-urgent evaluation and treatment to the outpatient setting and this may be influencing decisions surrounding discharge [25]. We found that the median readmission length of stay was two days, which suggests that a well-timed discharge without subsequent readmission may lead to more efficient utilisation of the healthcare system.

SSI (11%), bleeding (11%) and ileus (6%) were the next common reasons for readmissions in our cohort and also widely cited in the current literature on surgical readmissions [2,26]. A review of nearly 500,000 surgical admissions across nearly 350 hospitals in the USA found that SSI (19.5%), ileus (10.3%) and bleeding (4.9%) were the three most common reasons for readmission within 30 days of surgery [26]. 

Another study examining general surgical readmissions also noted the top two reasons for readmission to be a gastrointestinal complication (27.65%) and surgical infections (22.09%) [2]. Given the ubiquity of readmission for SSI, it is pertinent to highlight that half of our readmissions for SSI may have been avoided by following local surgical antibiotic prophylaxis guidelines and having adequately treated recognised SSIs during the index admission.

Although readmissions for life-threatening complications were not noted, more than one-fifth of our cohort received procedural intervention during readmission. This reflects poor outcomes for patients and the healthcare system and the preventability of readmissions need to be addressed. Due to the subjectivity in defining and assessing preventability, it is difficult to gauge how many readmissions are preventable. Reported rates of preventability range widely between 5% and 79% [27]. The proportion of very early readmissions that may be preventable is likely underrepresented and masked in the current literature and pointedly, recent Australian data have shown that one in eight readmissions within a day after discharge was preventable [12]. Similarly, a prospective cohort study showed that more readmissions within seven days of discharge were preventable than those between eight and 30 days after discharge [25]. 

Extensive research has gone towards identifying and measuring the success of interventions to reduce readmissions. These have been categorised as pre-discharge, post-discharge and bridging interventions that improve the success of the transition from hospital to home [28]. Regarding readmissions within the day after discharge, predictably, the hospital has been identified as a better location to intervene when compared with the home or the outpatient clinic [25]. Physician decision-making, inpatient processes and transitional care planning have been suggested as index admission factors and hospital factors that could be targeted [25]. Patients, however, are and should be as involved as healthcare professionals in any “good” discharge, and recurring themes that have been identified as contributing to readmission include 1) poor patient education, 2) poor understanding around discharge instructions and follow-up and 3) communication deficits between healthcare professionals and patients and carers [24,26,28]. Remedying this with interventions that are orientated towards patient engagement and empowerment and that promote patients’ and carers’ capacity for patient self-care have been shown to be most effective at reducing readmissions [29]. We support formal assessments of readiness for discharge that involve patients, carers and healthcare professionals to help reduce the risk of premature discharge and subsequent readmission [3].

Post-discharge interventions, if being undertaken, need to occur very soon after discharge as our results and those of others have shown that many readmissions occur early within the first month after discharge [5]. While no single intervention has been consistently shown to reduce the risk of readmissions, post-discharge telephone calls have been shown to be a common component of successful bundled interventions [29]. Some readmissions may also be able to be managed in the outpatient setting with community nursing and very early outpatient follow-up [26]. Given the ubiquity of SSI as a reason for readmission, we encourage earlier than traditional outpatient monitoring of wounds.

Close to a third of our cohort were readmitted after elective surgery and this offers the unique potential for intervention in the pre-admission or pre-operative phase. Risk factors for readmission can be categorised as patient-specific, clinician-specific and system-specific factors and the pre-admission or pre-operative phase is an ideal opportunity to address patient-specific factors [30]. This includes measures such as ensuring good blood glucose control to reduce post-operative SSI, referral to pain specialists for input on peri-operative pain management where appropriate and listening to and managing patients’ expectations.

Limitations of this study include bias related to the relatively small sample size, even though readmissions to our service were examined across five years. The study was also dependent on the accuracy of medical records. One of the issues faced in our analysis was subjectivity in defining and categorising the reason for readmission and in determining preventability. Two reviewers were used as opposed to one in order to mitigate some of this bias. This variability was also noted in the literature which was additionally clouded by heterogeneity in 1) the source of data on readmissions and 2) the time period across which readmission was measured [1,7,15]. Our results were derived from a single centre and so reflect local practices which may have limited applicability elsewhere due to the heterogeneity between hospitals. Indeed, research shows that some hospitals fare inherently worse than others in readmissions even across the same patients and so the most clinically relevant data may only be available from self-audit [8].

## Conclusions

Surgical readmissions within the first day after discharge are not uncommon. Pain, inadequate treatment of the index admission illness and SSI are common reasons for readmission within this time period. Targeting 1) better pain management on the day of discharge, 2) following local antibiotic prophylaxis guidelines and adequately treating recognised SSIs before discharge and 3) involving patients and carers in formal assessments regarding readiness for discharge may prevent these readmissions. Medical optimisation, patient education and management of expectations prior to elective surgery should also be vital steps taken to prevent readmissions. 

In auditing readmissions to the General Surgical service after surgery and after conservative management, this study provides more useful and realistic information for both clinicians and administrators. This study reinforces that surgical readmissions are distinct from medical and often related to well-described complications of surgery. Therefore, one approach to address readmissions across all patients is unlikely to be effective. Given that surgical readmission rates have been associated with higher-quality surgical departments, there should be a strong impetus for surgeons to measure and reduce readmission rates within their own hospitals. Finally, some readmissions will not be preventable and are the correct course of action for complications that should be treated in the inpatient setting.
